# Afu-Emi1 Contributes to Stress Adaptation and Voriconazole Susceptibility in Aspergillus fumigatus

**DOI:** 10.1128/spectrum.00956-23

**Published:** 2023-04-11

**Authors:** Jufang Tan, Heng Zhang, Yi Sun, Lujuan Gao

**Affiliations:** a Department of Neonatology, Jingzhou Hospital Affiliated to Yangtze University, Jingzhou, Hubei Province, China; b Department of Surgery, Jingzhou Hospital Affiliated to Yangtze University, Jingzhou, Hubei Province, China; c Department of Dermatology, Jingzhou Hospital Affiliated to Yangtze University, Jingzhou, Hubei Province, China; d Department of Dermatology, Zhongshan Hospital, Fudan University, Shanghai, China; e Department of Dermatology, Zhongshan Hospital (Xiamen), Fudan University, Xiamen, Fujian Province, China; f Xiamen Clinical Research Center for Cancer Therapy, Xiamen, Fujian Province, China; University of Guelph College of Biological Science

**Keywords:** *Aspergillus fumigatus*, *Afu-emi1*, voriconazole, *cyp51A*, stress adaptation, efflux pump, azoles, AFUA_1G07360

## Abstract

Invasive aspergillosis (IA) is the second most common invasive fungal disease and is associated with high mortality rates. Aspergillus fumigatus is the predominant causal agent of this life-threatening infection. Triazoles are still the cornerstone of antifungal treatment, and voriconazole remains the first-line choice. However, voriconazole resistance has been increasingly reported, which results in significantly higher mortality rates for IA and is particularly problematic. In the present study, we report the identification and functional study of a protein with previously unknown function that is encoded by the gene designated *Afu-emi1* (AFUA_1G07360). High-throughput gene replacement technology was applied to construct the knockout Δ*Afu-emi1* strain and a revertant strain. The MICs for azoles, including posaconazole, itraconazole, and voriconazole, were evaluated via the broth microdilution method and E-tests, which revealed that disruption of *Afu-emi1* resulted in 4-fold increased susceptibility to voriconazole. Colony growth in the presence of oxidants, namely, H_2_O_2_ and menadione, and osmotic pressure-altering agents, namely, NaCl and d-sorbitol, was measured. The *Afu-emi1* mutant strain exhibited a significant growth defect under oxidative and osmotic stress. The reactive oxygen species (ROS) production levels with or without voriconazole pretreatment were determined, and the *Afu-emi1* mutant strain exhibited significantly lower ROS production levels. The effects of *Afu-emi1* disruption on voriconazole susceptibility, growth under stress, and ROS production were restored in the revertant strain. In addition, the expression of *cyp51A*, *AfuMDR2*, *AfuMDR3*, *AfuMDR4*, and *cdr1b* in the Δ*Afu-emi1* strain was significantly reduced. In conclusion, deletion of the gene *Afu-emi1* resulted in increased voriconazole susceptibility, attenuated ability for oxidative and osmotic stress adaptation, decreased ROS production, and downregulation of *cyp51A, AfuMDR2*, *AfuMDR3*, *AfuMDR4*, and *cdr1b* expression, suggesting that Afu-Emi1 is an important regulator of stress adaptation and *cyp51A* and efflux pump expression in this medically important fungus.

**IMPORTANCE** Voriconazole is the first-line choice for IA, a life-threatening disease. Therefore, voriconazole resistance has become particularly problematic. Disruption of *Afu-emi1* resulted in increased susceptibility to voriconazole, a significant growth defect under oxidative and osmotic stress, and downregulation of target enzyme Cyp51A and efflux pump expression, suggesting that Afu-Emi1 is an important regulator of stress adaptation and *cyp51A* and efflux pump expression in this medically important fungus. Targeting Afu-Emi1 might help to enhance azole therapeutic efficacy and impede azole resistance.

## INTRODUCTION

Aspergillus fumigatus, one of the most ubiquitous saprophytic fungi, is the most common causal agent of life-threatening invasive aspergillosis (IA) (1, 2), which affects approximately 300,000 people per year worldwide (3). In addition, A. fumigatus is the major causative factor for chronic lung and sinus disease, allergic bronchopulmonary aspergillosis, and Aspergillus bronchitis (4). Azoles, including itraconazole (ITC), voriconazole (VRC), posaconazole (POS), and isavuconazole, which inhibit ergosterol biosynthesis, are the cornerstones of antifungal treatment against aspergillosis. Among azoles, VRC is the preferred first-line antifungal agent (5). However, azole resistance of both clinical and environmental strains has been reported worldwide and has continued to increase in frequency over the past decade; it has a significant impact on morbidity and mortality rates, thus increasing the medical burden (5).

VRC resistance in particular is a problematic issue, challenging clinical management of the disease (6). van der Linden and colleagues in Nijmegen, the Netherlands, reported that the case fatality rate for patients with IA caused by a TR34/L98H multiazole-resistant isolate was 88.0% in a small case series of 8 patients (7). Another retrospective multicenter cohort study reported a significantly higher mortality rate for IA associated with VRC resistance (23 of 37 patients [62%]), compared with VRC-susceptible IA (58 of 158 patients [37%]) (*P* = 0.0038) (8). In view of this, it is of great significance to develop new antifungal targets that would help to increase susceptibility to VRC and potentiate the effect of VRC.

Previously, Whaley et al. mentioned that deletion of CAGL0K05797g (*EMI1*) in Candida glabrata yielded a 4-fold increase in susceptibility to fluconazole (9). However, no further information was provided. In an effort to discover novel antifungal targets that would enhance the sensitivity of A. fumigatus to azoles, we identified a homologous protein with previously unknown function that was encoded by the gene designated AFUA_1G07360 (GenBank identification no. 3507742). NCBI sequence alignment revealed 51.06% similarity with a CAGL0K05797g-encoded protein in Candida glabrata and 48.94% similarity with an *EMI1* (early meiotic induction 1)-encoded protein in Saccharomyces cerevisiae. Therefore, AFUA_1G07360 was named *Afu-emi1*. However, there are few literature reports available regarding Emi1 in pathogenic fungi. Here, we describe the disruption of the putative protein Afu-Emi1 in A. fumigatus and show that disruption of *Afu-emi1* results in increased susceptibility to VRC, decreased growth under oxidative and osmotic stress, decreased reactive oxygen species (ROS) production, and reduction in the expression of *cyp51A*, *AfuMDR2*, *AfuMDR3*, *AfuMDR4*, and *cdr1b*.

## RESULTS

### Bioinformatic analysis of *Afu-emi1*.

**FIG 1:**
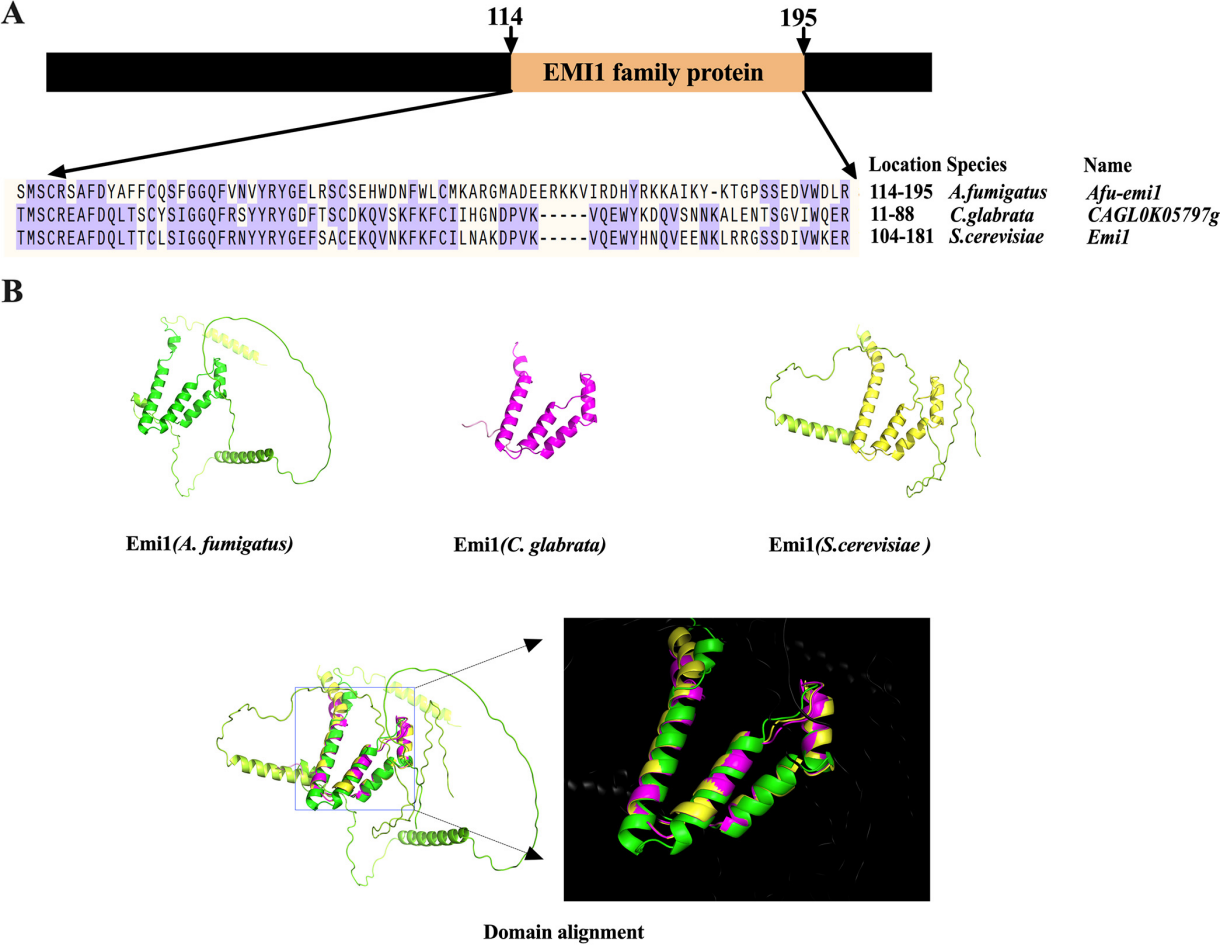
Bioinformatic analysis of *Afu-emi1*. (A) A BLAST search for the deduced amino acid sequence of AFUA_1G07360 protein revealed that the most familiar proteins and the corresponding genes are *emi1* in Candida glabrata (CAGL0K05797g) (51.06%) and S. cerevisiae (48.94%). (B) Domain alignment indicated that these proteins share an Emi1p domain with three α-helices.

The gene AFUA_1G07360 was located on chromosome 1 and consisted of 684 bases. The encoded protein contained 227 amino acids. A sequence homology search was performed. BLASTp screening for the deduced amino acid sequence of AFUA_1G07360 protein revealed that the most similar proteins and the corresponding genes are *emi1* in Candida glabrata (CAGL0K05797g) (51.06%) and S. cerevisiae (48.94%). In addition, the alignment indicated that these proteins share an Emi1p domain with three α-helices (Fig. 1). Accordingly, AFUA_1G07360 was named *Afu-emi1*.

### Effect of *Afu-emi1* on *in vitro* antifungal susceptibility profiles.

As shown in Table 1, the VRC MIC against a Δ*Afu-emi1* strain (0.0625 μg/mL) exhibited a 4-fold reduction, compared to that of wild-type (WT) and Δ*Afu-emi1*::*Afu-emi1^+^* strains (0.25 μg/mL). The MICs of ITC and POS were 1 μg/mL and 0.25 μg/mL, respectively, for all strains. The Etest results (Table 1; also see Fig. S1 in the supplemental material) revealed similar trends, compared with previous results determined with the broth microdilution method. Etest revealed that ITC MICs were 1.5 μg/mL for the WT and Δ*Afu-emi1*::*Afu-emi1^+^* strains and 1 μg/mL for the Δ*Afu-emi1* strain. Etest MICs for POS were 0.19 μg/mL for the Δ*Afu-emi1*::*Afu-emi1^+^* strain and 0.25 μg/mL for the WT and Δ*Afu-emi1* strains. Notably, Etest MICs for VRC were 0.094 μg/mL for the Δ*Afu-emi1* strain, 0.38 μg/mL for the WT strain, and 0.5 μg/mL for the Δ*Afu-emi1*::*Afu-emi1^+^* strain, demonstrating a dramatic increase in the susceptibility of the Δ*Afu-emi1* strain to VRC.

**TABLE 1:** Effects of *Afu-emi1* on *in vitro* antifungal susceptibility profiles

Strain	MIC (μg/mL)					
	Via broth microdilution			Via E-test		
	VRC	ITC	POS	VRC	ITC	POS
WT	0.25	1	0.25	0.38	1.5	0.25
Δ*Afu-emi1*	0.0625	1	0.25	0.094	1	0.25
Δ*Afu-emi1*::*Afu-emi1+*	0.25	1	0.25	0.5	1.5	0.19

### Effect of *Afu-emi1* on antioxidative stress and the production of intracellular ROS.

**FIG 2:**
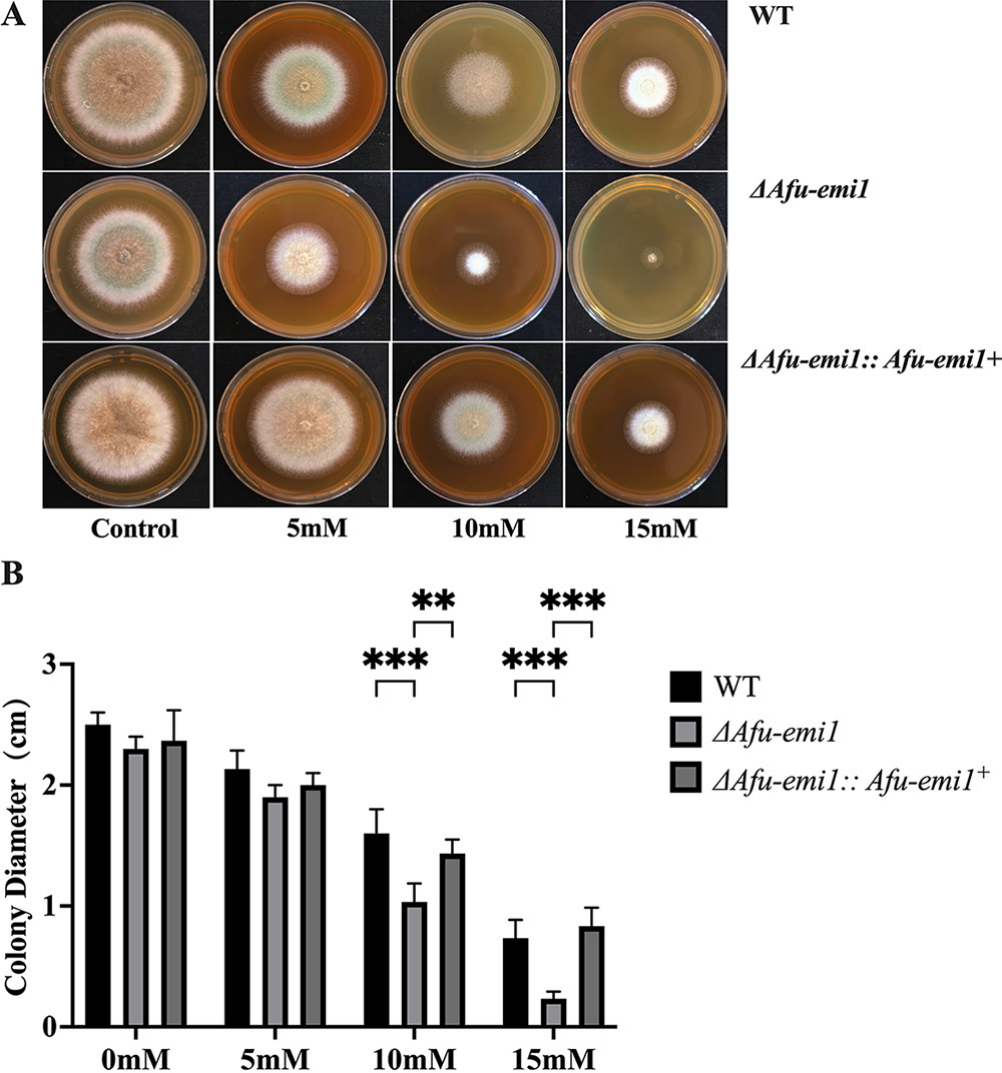
Growth of strains in the presence of menadione. (A) As the concentration of menadione increases, the growth of the Δ*Afu-emi1* strain is dramatically reduced. (B) Colony diameter evaluation revealed a significant growth defect of the Δ*Afu-emi1* strain at higher concentrations of menadione (10 and 15 mM). ***, *P* < 0.001; **, *P* < 0.01.

**FIG 3:**
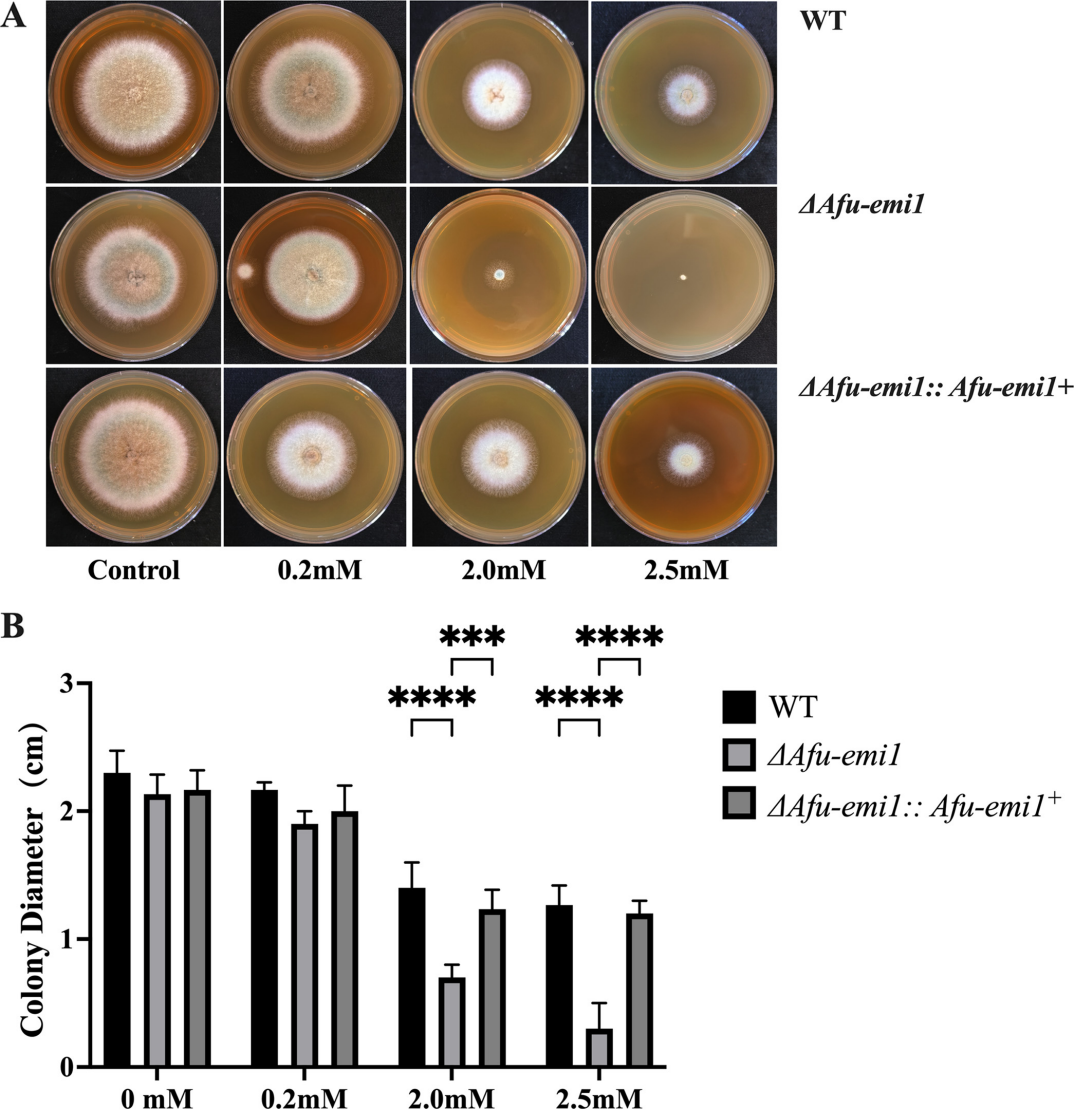
Growth of strains in the presence of H_2_O_2_. (A) As the concentration of H_2_O_2_ increases, the growth of the Δ*Afu-emi1* strain is dramatically reduced. (B) Colony diameter evaluation revealed a significant growth defect of the Δ*Afu-emi1* strain at higher concentrations of H_2_O_2_ (2 and 2.5 mM). ****, *P* < 0.0001; ***, *P* < 0.001.

**FIG 4:**
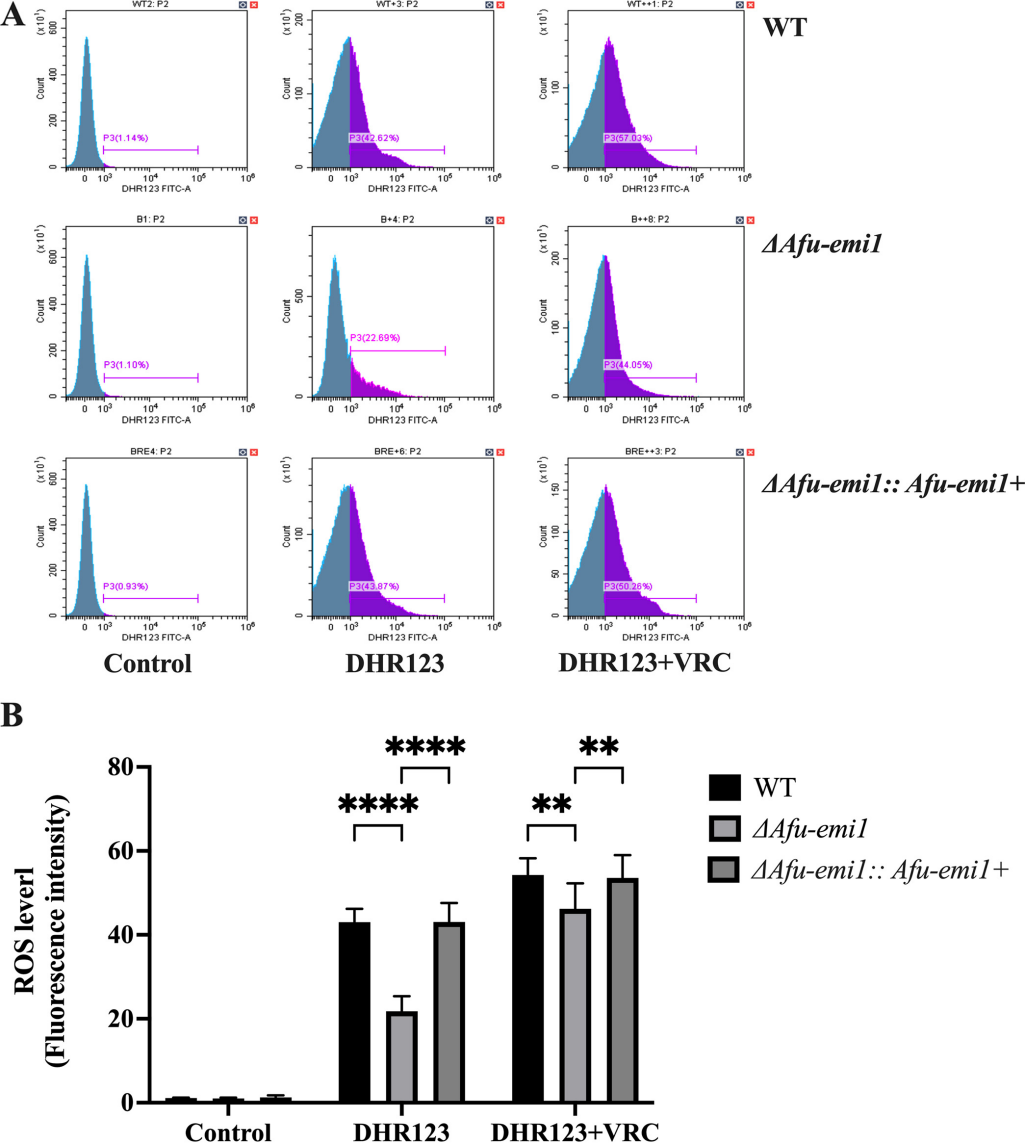
Intracellular ROS production levels. (A) The production of ROS by the Δ*Afu-emi1* strain with or without VRC was dramatically decreased, compared to the WT and Δ*Afu-emi1*::*Afu-emi1^+^* strains. (B) The ROS levels in the Δ*Afu-emi1* strain with or without VRC were significantly lower. ****, *P* < 0.0001; **, *P* < 0.01.

The growth of the Δ*Afu-emi1* strain was significantly reduced in the presence of increasing concentrations of menadione and H_2_O_2_, compared to the WT strain (*P* < 0.05) (Fig. 2 and 3). The growth defect of the Δ*Afu-emi1* strain caused by oxidative stress was restored by the reintroduction of *Afu-emi1* into the Δ*Afu-emi1* strain (Δ*Afu-emi1*::*Afu-emi1^+^* complementation strain). In addition, the production of ROS by the Δ*Afu-emi1* strain with or without VRC was significantly decreased, by 29.5% and 15.1%, respectively, compared to the WT strain (*P* < 0.05) (Fig. 4). However, the reduction of ROS was restored in the Δ*Afu-emi1*::*Afu-emi1^+^* strain (Fig. 4).

### Effect of *Afu-emi1* on antiosmotic stress.

**FIG 5:**
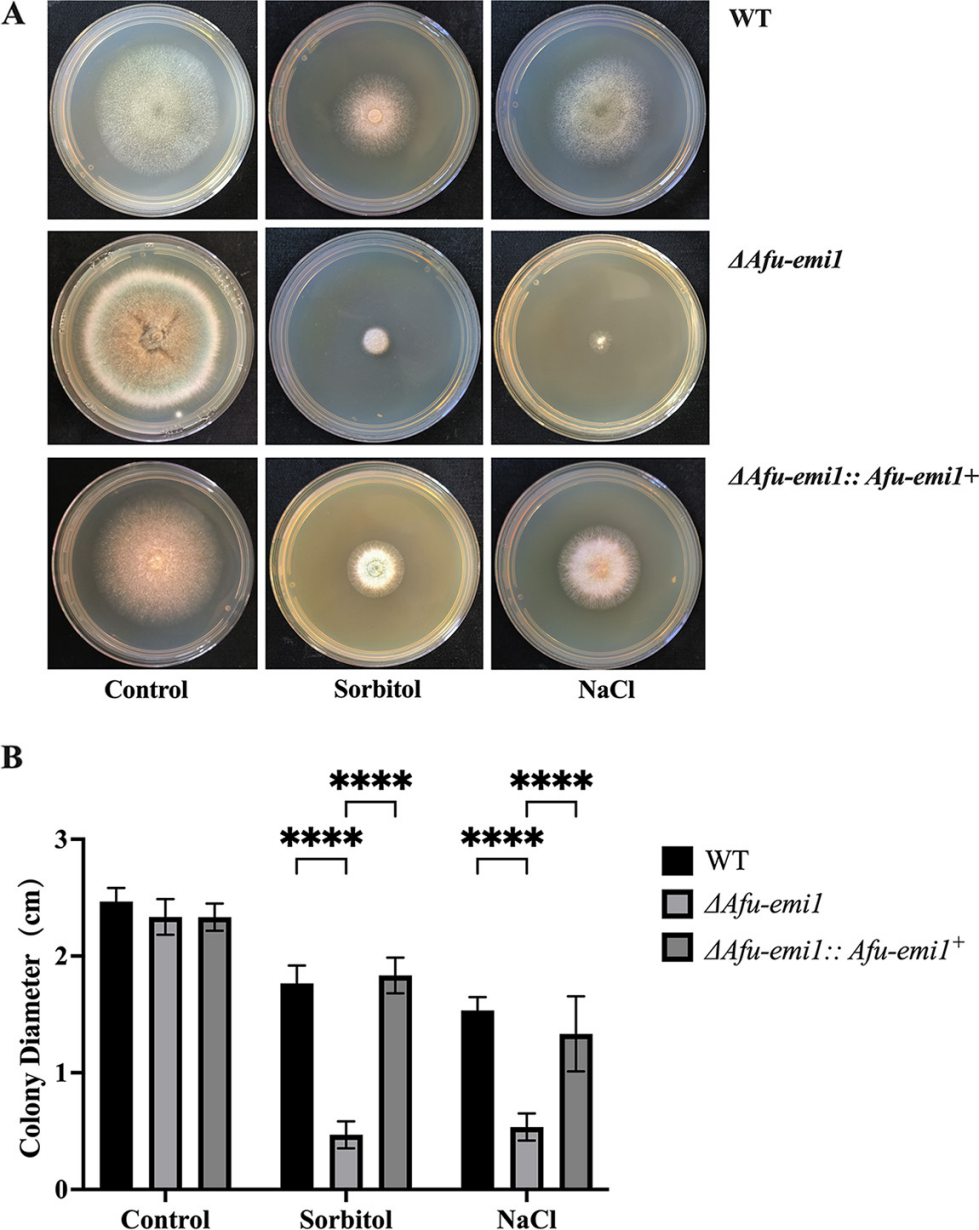
Growth of strains under osmotic stress. (A) In the presence of NaCl and sorbitol, the growth of the Δ*Afu-emi1* strain is dramatically reduced. (B) Colony diameter evaluation revealed a significant growth defect of the Δ*Afu-emi1* strain. ****, *P* < 0.0001.

The growth of the Δ*Afu-emi1* strain was significantly reduced in the presence of NaCl and sorbitol, compared with the WT strain (*P* < 0.05) (Fig. 5). However, the growth defect of Δ*Afu-emi1* due to osmotic stress was restored in the Δ*Afu-emi1*::*Afu-emi1^+^* strain (Fig. 5).

### Effect of *Afu-emi1* on gene expression.

**FIG 6:**
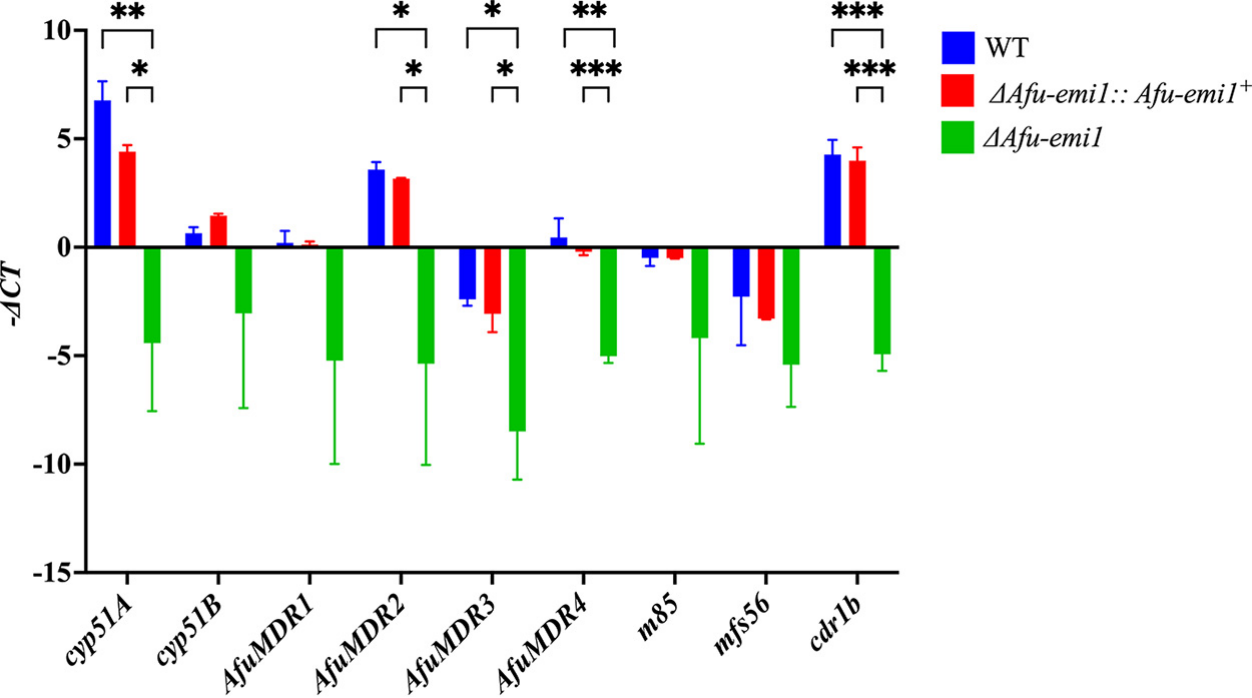
RT-PCR assay. The expression of *cyp51A*, *AfuMDR2*, *AfuMDR3*, and *cdr1b* in the Δ*Afu-emi1* strain was significantly reduced, compared to that in the WT strain and the revertant strain. ***, *P* < 0.001; **, *P* < 0.01; *, *P* < 0.05.

The expression of *cyp51A*, *AfuMDR2*, *AfuMDR3*, *AfuMDR4*, and *cdr1b* in the Δ*Afu-emi1* strain was significantly reduced, compared to that of the WT and Δ*Afu-emi1*::*Afu-emi1^+^* strains (*P* < 0.05) (Fig. 6). No significant difference was observed in the expression of *cyp51B*, *AfuMDR1*, *mfs56*, and *m85* (Fig. 6).

## DISCUSSION

The result of the present work demonstrated that disruption of *Afu-emi1* resulted in a 4-fold increase in VRC susceptibility, while the complemented strain showed VRC sensitivity comparable to that of the WT strain, suggesting that manipulation of *Afu-emi1* was responsible for these phenotypes and that *Afu-emi1* was required for normal VRC resistance. Overexpression and/or alterations of the target enzyme Cyp51 protein, decrease azole accumulation due to upregulation of efflux pump systems, including ATP-binding cassette (ABC) and major facilitator superfamily (MFS) efflux pumps, and oxidative stress adaptation (6, 10) are the underlying mechanisms associated with azole resistance/susceptibility. To expand our knowledge of the role of *Afu-emi1* in VRC susceptibility, we further investigated growth under stress, ROS production, and major gene expression in the absence of *Afu-emi1.*

It is well known that the ability of A. fumigatus to adapt quickly to various unfavorable environmental stresses, including osmotic or oxidative stress, extreme temperature, and nutrient limitation, is important for the pathogen to survive under long-term adverse environmental conditions and resist antifungal agents (11). Azole exposure leads to ROS production, which contributes to growth inhibition. Therefore, oxidative stress adaptation has been demonstrated to contribute to azole resistance (6). In the present study, deletion of *Afu-emi1* resulted in significantly reduced growth under oxidative stress with menadione and H_2_O_2_, indicating that the ability for oxidative stress adaptation was attenuated. The attenuated oxidative stress adaptation might be responsible for the increase in the susceptibility to VRC in the Δ*Afu-emi1* strain. Additionally, impaired growth under hyperosmotic stress with NaCl and sorbitol was documented in the Δ*Afu-emi1* strain, suggesting that the osmotic stress response, which is required for full virulence, was also impacted by Afu-Emi1 protein.

A previous study revealed that mitochondrial inhibition resulted in abolishment of ROS accumulation in A. fumigatus when it was exposed to azoles, which might lead to azole resistance (6). In our study, however, the ROS production levels with or without VRC were both significantly decreased in the Δ*Afu-emi1* strain, which showed a 4-fold decrease in susceptibility to VRC, suggesting that factors other than ROS production play roles in the VRC sensitivity of the Δ*Afu-emi1* strain.

In the absence of target gene mutations, azole resistance can also be driven by overexpression of the target enzyme Cyp51A, which increases the azole concentration necessary to inhibit fungal growth, and upregulation of efflux pump systems, including ABC transporters and MFS transporters, which decrease the intracellular drug concentration (6, 12). To decipher the mechanisms responsible for VRC sensitivity of the Δ*Afu-emi1* strain, the expression of *cyp51A*, *cyp51B*, *AfuMDR1*, *AfuMDR2*, *AfuMDR3*, *AfuMDR4*, *cdr1b*, *mfs56*, and *m85* was determined by real-time quantitative PCR (qPCR), which revealed significantly decreased expression of *cyp51A*, *AfuMDR2*, *AfuMDR3*, *AfuMDR4*, and *cdr1b* in the Δ*Afu-emi1* strain.

The *cyp51* genes encode lanosterol 14-demethylases, a key enzyme involved in ergosterol biosynthesis. The azole antifungals inhibit the ergosterol biosynthesis pathway by the act of inhibiting 14-demethylases. A. fumigatus presents two paralogues (*cyp51A* and *cyp51B*) of *cyp51*. It has been demonstrated that the expression of *cyp51A* correlates with azole susceptibility. Overexpression of *cyp51A* reduces azole susceptibility in Aspergillus spp. (13), whereas *cyp51B* was demonstrated to have no effect on azole susceptibility (14). Deletion of *Afu-emi1* resulted in decreased expression of *cyp51A*, which might contribute to the increased VRC susceptibility of the Δ*Afu-emi1* strain. Efflux pumps can pump intracellular toxins or drugs out of the cell and help to overcome intracellular toxin or drug accumulation; therefore, efflux pumps have important roles in antifungal susceptibility and virulence. Members of the ABC transporter superfamily export substrate by coupling transport with ATP hydrolysis, while MFS members remove compounds with proton gradients (15). In A. fumigatus, ABC transporters Cdr1B, AfuMDR1, AfuMDR2, AfuMDR3, AfuMDR4, AbcA to AbcE, and AtrF and MFS transporters MfsA to MfsC have been reported in several studies and have been shown to be associated with increased azole resistance (13, 16–22). It was reported that VRC exposure in a clinical azole-susceptible isolate resulted in induction of both ABC transporters, such as Cdr1b, and MFS transporters (23). Notably, overexpression of the *AfuMDR4* pump was found in a biofilm phenotype of A. fumigatus resistance to VRC (22). In the present study, the expression of *AfuMDR2*, *AfuMDR3*, *AfuMDR4*, and *cdr1B* was significantly reduced when *Afu-emi1* was disrupted, which might abrogate the possible effect of VRC exposure on the pump expression and thereby explain the increased VRC susceptibility of the knockout strain. Although our findings provide the first evidence that Afu-Emi1 has a regulatory role in the expression of *cyp51A* and efflux pumps, further studies are warranted to elucidate the correlation between this expression profile and VRC susceptibility in the Δ*Afu-emi1* strain.

In summary, deletion of the gene coding for the protein homologous to S. cerevisiae EMI1 resulted in increased VRC susceptibility, attenuated ability for oxidative and osmotic stress adaptation, decreased ROS production with or without VRC, and downregulation of *cyp51A*, *AfuMDR2*, *AfuMDR3*, *AfuMDR4*, and *cdr1b* expression in A. fumigatus, suggesting that Afu-Emi1 is an important regulator of stress adaptation and *cyp51A* and efflux pump expression in this medically important fungus and that Afu-Emi1 could be a promising antifungal target that could enhance the effect of VRC.

## MATERIALS AND METHODS

### Strains, plasmids, chemical agents, media, and growth conditions.

A. fumigatus strain AF293, which contained the complete A. fumigatus DNA sequence, was used as the template for preparing the knockout products and WT strain for phenotype studies. The A. fumigatus Δ*KU80* pyrG− strain (A1160, obtained from the Fungal Genetics Stock Center), which is auxotrophic for uracil/uridine and possesses an increased rate of homologous recombination, was used as the host strain for transformation. Plasmid pLAX223, containing *pyrG* for uracil/uridine synthesis, and plasmid pCT74, containing hygromycin B phosphotransferase (*hph*) for inducing hygromycin B resistance, were obtained from Hunan Fenghui Biotechnology Co., Ltd. (China). Commercially available antifungal chemicals, including VRC, ITC, POS, and dihydrorhodamine-123 (DHR-123) (CAS no. 109244-58-8), were obtained from Sigma-Aldrich (Shanghai) Trading Co. Ltd. (China). Pectinase and glucanase were obtained from Sunson Industry Group, Co. Ltd. (China).

The A. fumigatus strains used were maintained on Sabouraud dextrose agar (SDA). All A. fumigatus strains were grown at 37°C. Conidiospores that had been freshly grown for 3 days were suspended in sterile distilled water containing 0.03% Triton X-100 and were used for the experiments. Selection of the correct clones occurred on Czapek’s agar (CZA) lacking uridine/uracil or supplemented with hygromycin B (200 mg/L). Plasmid DNA was extracted after culturing at 37°C for 16 h on LB medium (1% tryptone, 0.5% yeast extract, 1% NaCl, and 2% agar for solid cultures) supplemented with 100 μg/mL ampicillin.

### Construction of *Afu-emi1* mutant strains.

A gene replacement cassette was generated to construct the deletion mutant Δ*Afu-emi1*. DNA manipulation was performed according to standard laboratory procedures. Genomic DNA (gDNA) was extracted from AF293 using the MolPure fungal DNA kit (Yeasen Biotechnology, Shanghai, China). Plasmid DNA was extracted using a MolPure plasmid minikit (Yeasen Biotechnology). To generate a replacement cassette for *Afu-emi1* (AFUA_1G07360 [GenBank identification no. 3507742]), the 5′ and 3′ flanking regions were amplified using primers P1/P2 and P3/P4, respectively (see Table S1 in the supplemental material). The selection maker *pyrG* was amplified from pLAX223 by using primers pyrG-F and pyrG-R (see Table S1). The 2×Hieff Canace PCR Master Mix (Yeasen Biotechnology) was used to amplify the related genes from gDNA or plasmid. These flanking regions along with a *pyrG* fragment were subsequently fused via PCR with primers P5 and P6 (see Table S1) (24), verified by agarose gel electrophoresis (approximately 4.0 kb), and purified by cutting the gel for subsequent application. The product obtained was used as the gene replacement cassette.

A 150-μL aliquot of an A. fumigatus A1160 conidial suspension was pipetted at the concentration of 1 × 10^9^ CFU/mL and inoculated into a 250-mL sterile flask containing 50 mL of Sabouraud broth (SAB) supplemented with uracil. The flask was incubated on a shaker at 130 rpm for 16 h at 37°C. A sterile Miracloth obtained from Sigma-Aldrich (Shanghai) Trading Co., Ltd., was used to filter and collect the mycelia, which were then treated with a protoplast solution composed of pectinase and glucanase and were incubated at 30°C for 4 h. Finally, KCl/CaCl_2_ solution was used to gradually resuspend the A. fumigatus protoplasts. The constructed *Afu-emi1* gene replacement cassette was mixed with protoplast to complete transformation, and the transformants were selected on CZA lacking uridine/uracil. Precise recombination and integration were confirmed by PCR using primers P1 and Kasel-R (see Table S1).

### Complementation of *Afu-emi1*.

The Δ*Afu-emi1*::*Afu-emi1^+^* transformants were reconstituted via transformation of protoplasts as described above. The protoplasts of Δ*Afu-emi1* strains were prepared as described above. The flanking and encoding regions of *Afu-emi1* were amplified from gDNA of AF293 using primers AIM-F and AIM-R. The *hph* fragment was amplified from the pCT74 plasmid vector with primers HPH-F and HPH-R (see Table S1). Subsequently, the *Afu-emi1* fragment and *hph* fragment were fused via fusion PCR with primers AIM-F and HPH-F (see Table S1) and verified by agarose gel electrophoresis (approximately 5.0 kb). The product obtained was used as the reply carrier and further mixed with Δ*Afu-emi1* protoplast to complete transformation. The transformants were selected on CZA supplemented with hygromycin B (200 mg/L). PCR validation was performed with primers Kasel-F2 and Kasel-R2 (see Table S1).

### Antifungal susceptibility testing.

*In vitro* susceptibilities to ITC, VRC, and POS of the WT AF293, Δ*Afu-emi1*, and Δ*Afu-emi1*::*Afu-emi1^+^* strains were determined according to the CLSI M38-A2 method (25). Conidia harvested from cultures that had been grown for 7 days on SDA were suspended in sterile distilled water containing 0.03% Triton X-100 and were diluted to a concentration of 1 × 10^6^ to 5 × 10^6^ spores/mL, which was than diluted 100 times in RPMI 1640 medium to achieve a suspension 2-fold more concentrated than the density needed or approximately 1 × 10^4^ to 5 × 10^4^ spores/mL. A 96-well plate was inoculated with 100 μL of the inoculum suspension and 100 μL of the serial dilution of the tested drugs. The working concentration ranges of tested drugs were 0.0625 to 4 μg/mL. Interpretation of results was performed after incubation at 35°C for 48 h. The MICs were determined as the lowest concentration resulting in complete inhibition of growth (25). Etest-mediated susceptibility testing (AB bioMérieux, Durham, NC, USA) for ITC, VRC, and POS was performed in accordance with the manufacture’s instructions. Candida parapsilosis ATCC 22019 was included to ensure quality control. All experiments were conducted in triplicate.

### Oxidative stress test.

Two kinds of oxidants, i.e., H_2_O_2_ and menadione, were added to SDA at different concentrations, namely, 0, 0.2, 2.0, and 2.5 mM for H_2_O_2_ and 0, 5, 10, and 15 mM for menaquinone. A 1-μL aliquot of conidial suspensions of WT, Δ*Afu-emi1*, and Δ*Afu-emi1*::*Afu-emi1^+^* strains at a concentration of 1 × 10^4^ CFU/mL was dropped in the center of the SDA and incubated at 37°C for 48 to 72 h. The diameters of the colonies were measured. All experiments were conducted in triplicate.

### Determination of intracellular ROS levels.

Conidial suspensions *of* Δ*Afu-emi1*, Δ*Afu-emi1*::*Afu-emi1^+^*, and WT strains were resuspended in 10 mL of SAB to achieve a concentration of 3 × 10^6^ to 5 × 10^6^ CFU/mL. A total of three groups were included for each strain studied, namely, a DHR-123 (5 μg/mL)-treated group, a DH123 (5 μg/mL)- and VRC (0.125 μg/mL)-treated group, and a control group. DHR-123 is an uncharged and nonfluorescent ROS detector that penetrates the cell membranes; it is oxidized to rhodamine-123 by intracellular ROS and emits bright-green fluorescence. The fluorescence intensity can be quantitatively detected by flow cytometry. Subsequently, conidial suspensions were incubated at 37°C and 130 rpm for 30 min. The spores were washed twice with 1× phosphate-buffered saline (PBS), resuspended in SAB, incubated at 37°C and 130 rpm for 1 h, further centrifuged, resuspended in PBS, and detected by flow cytometry (excitation wavelength, 488 to 505 nm; emission wavelength, 515 to 575 nm). Finally, 10,000 cells were collected, and the percentage of cells that produced fluorescence was calculated. All experiments were conducted in triplicate.

### Osmotic pressure test.

Different osmotic pressure-altering agents (0.8 mol/L NaCl and 1.2 mol/L d-sorbitol) were added to the SDA. Conidial suspensions of the Δ*Afu-emi1*, Δ*Afu-emi1*::*Afu-emi1^+^*, and WT strains at a concentration of 1 × 10^4^ CFU/mL were prepared. A 1-μL aliquot of suspension was dropped at the center of the SDA and incubated at 37°C for 48 to 72 h. The diameters of the colonies that grew on the plates were photographed and then measured. All experiments were conducted in triplicate.

### Real-time qPCR.

The expression of *cyp51A*, *cyp51B*, *AfuMDR1*, *AfuMDR2*, *AfuMDR3*, *AfuMDR4*, *cdr1b*, *mfs56*, and *m85* was determined by real-time qPCR. The Δ*Afu-emi1*, WT, and Δ*Afu-emi1*::*Afu-emi1^+^* strains were cultured on SDA for 2 days. Total RNA was extracted using the TRIeasy LS total RNA extraction reagent (Yeasen Biotechnology) and transcribed to cDNA via the Hifair II first-strand cDNA synthesis supermix for qPCR (gDNA Digester Plus) (Yeasen, Biotech Shanghai, China) according to the manufacturer’s instructions. The RT-qPCR was performed by using Hieff qPCR SYBR green master mix (Yeasen Biotechnology) in an ABI 7500 reverse transcription real time-PCR system. The primers used for RT-qPCR are shown in Table S2 in the supplemental material. The levels of each gene were calculated with the 2^−ΔΔ^*^CT^* method (26). The relative expression levels were normalized to the level of the internal control actin gene, where Δ*C_T_* of target gene = CT of target gene − CT of actin. Experiments were performed in triplicate.

### Statistical analysis.

Data were presented as the mean ± standard deviation (SD). Graph production, data distribution, and statistical analyses were performed using GraphPad Prism. After ensuring that the data conformed to a normal distribution, before and after data transformation, analysis of variance (ANOVA) and *t* tests were used to investigate significant differences between independent groups. Differences were considered significant at *P* values of <0.05.
