# Supplementary material for: Afu-Emi1 Contributes to Stress Adaptation and Voriconazole Susceptibility in Aspergillus fumigatus
Source: Microbiol Spectr. 2023 Apr 11;11(3):e00956-23. doi: 10.1128/spectrum.00956-23 (PMC10269808; doi:10.1128/spectrum.00956-23)
Supplement: Supplemental file 1 — Supplemental material. Download spectrum.00956-23-s0001.pdf, PDF file, 2.1 MB [file spectrum.00956-23-s0001.pdf]

Table S1. Primer used for gene manipulation

Primers	Sequences (5'–3')	Function
P1	ATGGTGAATCCGTGCATACAGTTC	Amplification upstream fragment of *Afu-emi1*
P2	TAGTTCTGTTACCGAGCCGGGTCCTGTGGATCGAGTTATTCACGT	
P3	GCTCTGAACGATATGCTCCAACCATCCCTCGCTTTTGGGCCCTGTCT	Amplification downstream fragment of *Afu-emi1*
P4	CTGATCGTCGTGACACTACTATTCG	
P5	TGGCCTTCCAATGACCCGACATACT	Fusion PCR for knockout cassette
P6	CTGATCGTCGTGACACTACTATTCG	
pyrG-R	CCGGCTCGGTAACAGAACTACCGCAGACAATGCTCTCTATC	Amplification of *pyrG* fragment
pyrG-F	GTTGGAGCATATCGTTCAGAGCAATACCGTTACACATTTCCA	
Kasel-R	CATGTAGAGGGTGCGGAG	Verify that the filter tags are inserted
HPH-R	GTTGGAGCATATCGTTCAGAGCCCTCTAAACAAGTGTACCTGTGC	Amplification of *hph* fragment
HPH-F	CCGGCTCGGTAACAGAACTATTCTATTTGTGTTTGATCGAGACC	
AIM-F	TCCCATAGGGGCGAAATAGAGTG	Amplification of flanking and encoding regions of *Afu-emi1*
AIM-R	TAGTTCTGTTACCGAGCCGGAGCAGCTATGCATGTTTGAGAAT	
Kasel-F2	AGAATCTCGTGCTTTCAGCTTC	Verify that the target gene is inserted
Kasel-R2	CATTGTCCGTCAGGACATTGTTG	

Table S2. Primers used in RT-qPCR

Primers	Sequence(5’–3’)
cyp51AqPCR-F	TGCAGAGAAAAGTATGGCGA
cyp51AqPCR-R	CGCATTGACATCCTTGAGC
cyp51BqPCR-F	AGCAGAAGAAGTTCGTCAAATAC
cyp51BqPCR-R	TCGAAGACGCCCTTGTG
MDR1qPCR-F	TTCCCTTGTTCACAATTCTCTTCG
MDR1qPCR-R	TGACATAGACTGTGACAAACTCG
MDR2qPCR-F	TTTAGCTCCACCGGGTTTG
MDR3qPCR-R	ATATCCATCCCCCAGGC
MDR4qPCR-F	TATGGCTTAGTTTGTTTGTGTCACCGA
MDR4qPCR-R	AGAGCAATTCGTTGCTTCTG
cdr1BqPCR-F	GAGTGCGTACGATGTATTCGAC
cdr1BqPCR-R	GGCAGGACTGGTGAGAGAAG
MFS56qPCR-F	GGGGGTATGGTAATTGGAGGT
MFS56qPCR-R	AAGAAGCGCAGACCATCG
M85qPCR-F	GCCAAGCACTATGAGCCTTC
M85qPCR-R	GCCCAGTTCCTTCCAGATAA
Actin-F	CTGTGCACATTGTCGCCAGGG
Actin-R	GTCCAGATTAAGCTGTCGCGC

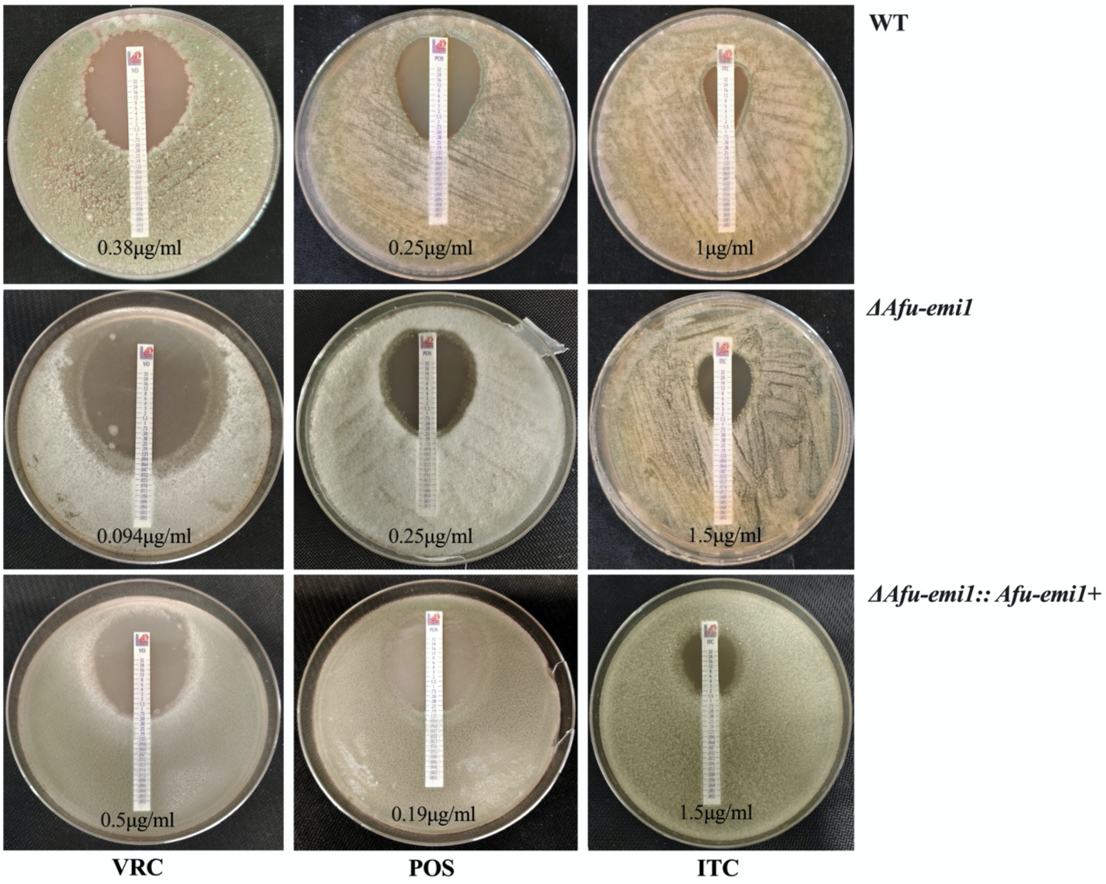

Fig S1. E-test results. The MICs of ITC and POS were comparable among strains, whereas the MIC of VRC against *ΔAfu-emi1* were dramatically decreased as compared to WT and *ΔAfu-emi1:: Afu-emi1+*.
